# Association of depressive symptoms with chronic liver disease among middle-aged and older adults in China

**DOI:** 10.3389/fpsyt.2023.1273754

**Published:** 2023-10-24

**Authors:** Jingke Zeng, Xiaohuan Lai, Shuigen Wang, Dijing Zeng, Jiangmin Ye, Chunhua Huang, Minhua Liu, Wenjuan Zhang, Hui Xu

**Affiliations:** ^1^Department of Hepatology, Ganzhou Institute of Liver Diseases, The Fifth People’s Hospital of Ganzhou, Ganzhou, Jiangxi, China; ^2^Longnan First People's Hospital, Ganzhou, Jiangxi Province, China; ^3^School of Basic Medicine, Gannan Medical University, Ganzhou, Jiangxi, China; ^4^Big Data Center, Beijing Children's Hospital, Capital Medical University, National Center for Children's Health, Beijing, China

**Keywords:** depressive symptoms, chronic liver disease, middle-aged and older adults, China, cohort study

## Abstract

**Background:**

The relationship between depressive symptoms and chronic liver disease (CLD) is still unclear. We aimed to determine whether depressive symptoms are associated with CLD in a large population sample.

**Methods:**

The data was from the China Health and Retirement Longitudinal Study (CHARLS), an ongoing nationally representative prospective cohort study. Depressive symptoms were assessed with the catchment-area epidemiology survey-depression (CES-D). CLD was identified by the patient’s self-report about a physician’s diagnosis at each visit. Multi-adjusted logistic regression and Cox regression models were used.

**Results:**

A total of 14,995 participants (53.1% female; mean age: 58.85 ± 9.87 years) and 13,405 participants (53.64% female; mean age: 58.58 ± 9.69 years) were included in the cross-sectional and longitudinal analyses, respectively. In the cross-sectional analysis, the odds ratio of CLD in patients with moderate and severe depressive symptoms were 1.46 [95% confidence interval (CI), 1.16–1.83] and 1.78 (95% CI, 1.23–2.56) than those with none/mild depressive symptoms, respectively. In the longitudinal analysis, compared to participants with none/mild depressive symptoms, the hazard rates of CLD in those with moderate and severe depressive symptoms were 1.65 (95%CI, 1.33–2.03) and 1.80 (95%CI, 1.24–2.60). And the 50th percentile difference of time (years) at the incidence of CLD in participants with moderate and severe depressive symptoms were − 0.83 (95%CI, −1.18, −0.49) and − 0.96 (95%CI, −1.56, −0.35), respectively.

**Conclusion:**

Elevated depressive symptoms were associated with an increased risk of CLD among middle-aged and older adults in China.

## Introduction

Chronic liver disease (CLD) including chronic hepatitis B, alcoholic hepatitis, non-alcoholic steatohepatitis and other hepatitis, is a major public health issue worldwide with significant morbidity and mortality. According to global reports on chronic liver disease research, there are more than 1,600 million people with CLD (1, 2). About 20% population is affected by CLD in China (3, 4). Cirrhosis and other complications of CLD cause about 20,000 deaths each year, accounting for 3.5% of global mortality(2018). CLD not only reduces the quality of life of patients, but also creates a heavy economic burden (5). Therefore, it is necessary to identify modifiable risk factors for CLD in order to develop effective prevention strategies.

Depressive symptoms are common in middle-aged and older adults (6), and 30–40% of the population aged 45 years and above worldwide have depressive symptoms (7, 8). Previous studies have found that depressive symptoms are one of the important risk factors for a variety of adverse health outcomes (9–12), which could increase the risk of chronic physical diseases, such as cardiovascular disease, stroke, metabolic syndrome, and CLD. Several cross-sectional and longitudinal studies have shown that depression may be associated with an increased risk of CLD (10, 13, 14). However, the extent of the association has varied widely. Therefore, to reduce the risk of CLD, it is important to understand its association with depressive symptoms.

A few studies demonstrated a positive relationship between depressive symptoms and nonalcoholic fatty liver disease (NAFLD). In contrast, research to study the potential relationship between depressive symptoms and other CLD is scarce. Based on the current literature (10, 11), we supposed that depressive symptoms, in addition to increasing the risk of NAFLD, may also be a risk factor for other CLD. Therefore, based on the cross-sectional and longitudinal studies, we aimed to explore the association between depressive symptoms and CLD in middle-aged and elderly Chinese.

## Methods

### Study population

The study population was obtained from the CHARLS, an ongoing nationwide cohort study conducted by the National School of Development at Peking University. Details of the study design and methodology regarding the CHARLS have been provided previously (15). In brief, participants aged 45 and above were recruited from 450 communities and administrative villages distributed in 28 provinces of China through multistage probability sampling in 2011. And all participants were followed every 2-3 year in 2013, 2015 and 2018, respectively. Information on demographic, sociological, and health status was collected using a standardized questionnaire at each time. The CHARLS was approved by the Institutional Review Board of Peking University (IRB00001052-11015). All participants signed the informed consent and repository consent that allowed their data to be shared after a detailed presentation of the risks and benefits associated with study participation.

**Figure 1:**
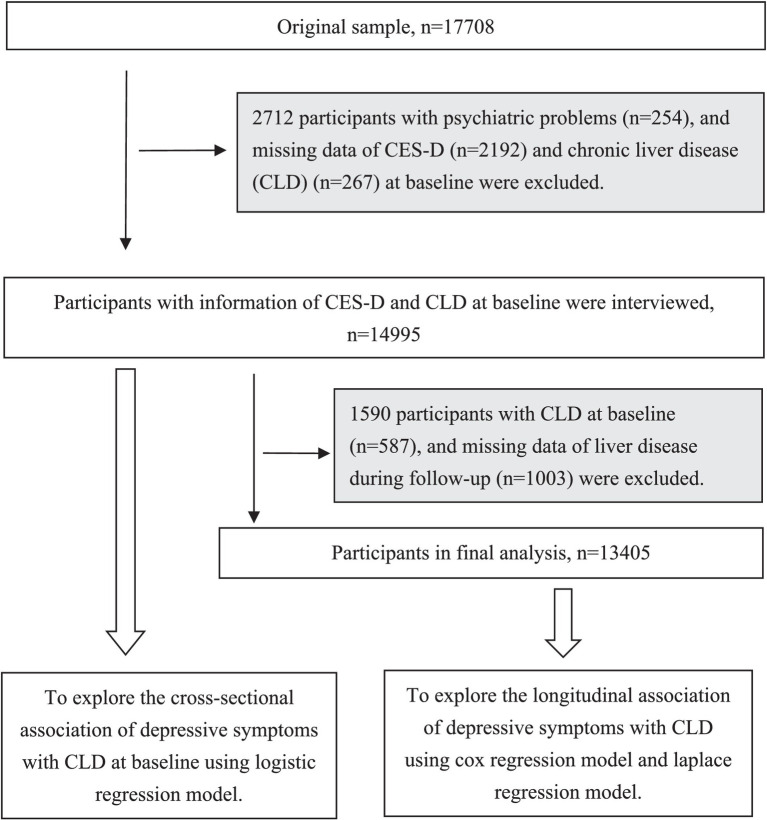
Flowchart of study participants.

A total of 17,708 participants were recruited at baseline. We excluded 2,567 participants with emotional, nervous, or psychiatric problems or depression (*n* = 254), and missing data on the Center for Epidemiologic Studies Depression Scale (CES-D; *n* = 2,192) and CLD (*n* = 267) at baseline. Then, 4,515 participants were available for the cross-sectional study. Subsequently, we additionally excluded 1,590 participants with CLD at baseline (*n* = 522) and missing data on the CLD during the follow-up (*n* = 1,003), leaving 13,405 individuals for the longitudinal analysis. The detailed flowchart of the sample selection process is shown in Figure 1.

### Assessment of depressive symptoms

Depressive symptoms were measured using CES-D which was a validated rating scale and had been widely used to assess depressive symptoms in the Chinese population (16, 17). The CES-D includes 10 items that refer to depressive symptoms during the last week. The score of each item ranges from 0 to 3 points: rarely or none of the time (<1 day), some or a little of the time (1–2 days), occasionally or a moderate amount of the time (3–4 days), most or all of the time (5–7 days). And the 10 items were: (1) bothered by things that do not usually bother you, (2) had trouble in keeping mind, (3) felt depressed, (4) felt everything was an effort, (5) felt hopeful about the future, (6) felt fearful, (7) sleep was restless, (8) felt happy, (9) felt lonely, and (10) could not get going. Before summing item scores, the scores of items 5 and 8 need to be reversed. The total score of CES-D was from 0 to 30, with a higher score indicating more depressive symptoms. As a previous study (18), depressive symptoms are divided into three categories: none/mild (0–9 points), moderate (10–20 points) and severe (21–30 points).

### Assessment of chronic liver disease

CLD were identified according to the patient’s self-report about a physician’s diagnosis at each visit. CLD included viral hepatitis, autoimmune hepatitis, primary biliary cirrhosis and primary sclerosing cholangitis, but except fatty liver, tumors, and cancer. And the condition of participants’ CLD was defined as yes and no.

### Assessment of potential confounders

Data of covariates including age, sex, education, marital status, residence, body mass index (BMI), smoking status, drinking status, hypertension, diabetes, stroke, heart disease (including heart attack, coronary heart disease, angina, congestive heart failure, or other heart problems), kidney disease, and dyslipidemia were collected at baseline. Education was categorized into illiterate, primary school and below, or middle school and above. Marital status was classified into two groups: married and another marital status (never married, separated, divorced, and widowed). BMI was calculated as weight in kilograms divided by height in meters squared (kg/m^2^). Both smoking and alcohol consumption were categorized as never, former, and current groups. Chronic diseases including hypertension, diabetes, stroke, heart disease, kidney disease, and dyslipidemia were self-reported and dichotomized as yes and no.

### Statistical analysis

Differences in characteristics among the two groups of participants with or without liver disease in the cross-sectional study and the three groups of participants with none/mild, moderate or severe depressive symptoms in the longitudinal study were evaluated using a one-way analysis of variance or Kruskal-Walli’s test for continuous variables, and Chi-square tests for categorical variables.

To examine the cross-sectional association of depression symptoms (continuous and categories) with CLD at baseline (wave 1), the logistic regression model was used to calculate the Odds ratio (OR) with 95% confidence intervals [Cls]. To examine the longitudinal association between depression symptoms (continuous and categories) and CLD, the Cox proportional hazards regression model was used to calculate the hazard ratios (HRs) with 95% Cls. And the laplace regression model was used to estimate the 50th percentile difference (PDs) in CLD onset time in the three groups (participants with none/mild, moderate or severe depressive symptoms). When depressive symptoms were used as a continuous variable, each point change of OR, HR and 50th PDs of CLD responded to a unit change of depressive symptoms. When depressive symptoms were used as a categorical variable (tertile), the points change in the OR, HR and 50th PDs of CLD were corresponding to the moderate or severe depressive symptoms in comparison to the none/mild. The proportional hazards assumption was tested before the Cox regression model was employed and no violation of the assumption was observed. All three models were estimated in 3 ways: age, sex, education, marital status, and residence were adjusted in model 1; BMI, smoking, alcohol consumption, and variables in model 1 were adjusted in model 2; hypertension, diabetes, stroke, heart disease, kidney disease, dyslipidemia, and variables in model 2 were adjusted in model 3.

In the sensitivity analysis, the difference of characteristics between individuals having the data of covariates and those lacking the data was analyzed. Multiple imputations by chained equation was used to impute data for 3,374 participants in the cross-sectional study and 2,665 participants in the longitudinal study with missing data of covariates including residence, BMI, smoking status, alcohol consumption, hypertension, diabetes, kidney disease, dyslipidemia. Furthermore, the stratified analysis by sex, smoking, and alcohol consumption, BMI in the longitudinal associations of depressive symptoms with CLD and the 50th PDs in CLD onset time was also performed.

All analyses were performed with Stata SE, version 15.0 (Stata Corp LP., College Station, Texas, United States), and statistical significance was defined as two-tailed *p* values less than 0.05.

## Results

### Characteristics of the cross-sectional study population

A total of 14,995 participants (53.1% female; mean age: 58.85 ± 9.87 years) were included in the cross-sectional analysis. Compared with participants without CLD, those with CLD were more likely to be male, smokers, drinkers, and have higher education, hypertension, diabetes, kidney disease, dyslipidemia, and CES-D. The difference between the two groups in age, marital status, residence, and BMI was not significant (Table 1).

**Table 1:** Baseline characteristics of the cross-sectional study population by chronic liver disease (CLD) (*n* = 14,995).

Characteristics	Total	Without CLD	With CLD	*P*
	N = 14,995	N = 14,408	N = 587	
Age (years)	58.85 ± 9.87	58.85 ± 9.90	58.87 ± 9.07	0.950
Female	7,962 (53.10)	7,683 (53.32)	279 (47.53)	0.010
Education level				0.012
Illiterate	3,944 (26.30)	3,822 (26.53)	122 (20.78)	
Primary school and below	5,947 (39.66)	5,686 (39.46)	261 (44.46)	
Middle school and above	5,097 (33.99)	4,893 (33.96)	204 (34.75)	
Marital status				0.340
Married	13,175 (87.86)	12,648 (87.78)	527 (89.78)	
Other	1818 (12.12)	1758 (12.20)	60 (10.22)	
Residence				0.460
Rural	6,878 (45.87)	6,598 (45.79)	280 (47.70)	
Urban	2,257 (15.05)	2,165 (15.03)	92 (15.67)	
BMI	23.53 ± 3.91	23.51 ± 3.91	23.84 ± 3.87	0.072
Smoking status				<0.001
Never	9,120 (60.82)	8,799 (61.07)	321 (54.68)	
Ever smoker	1,342 (8.95)	1,263 (8.77)	79 (13.46)	
Current smoker	4,530 (30.21)	4,343 (30.14)	187 (31.86)	
Alcohol consumption				<0.001
Never drinker	8,912 (59.43)	8,595 (59.65)	317 (54.00)	
Former drinker	1,234 (8.23)	1,161 (8.06)	73 (12.44)	
Current drinker	4,846 (32.32)	4,649 (32.27)	197 (33.56)	
Hypertension	3,651 (24.35)	3,472 (24.10)	179 (30.49)	<0.001
Diabetes	856 (5.71)	799 (5.55)	57 (9.71)	<0.001
Kidney disease	947 (6.32)	822 (5.71)	125 (21.29)	<0.001
Dyslipidemia	1,386 (9.24)	1,291 (8.96)	95 (16.18)	<0.001
CES-D (continuous)	8.33 ± 6.31	8.25 ± 6.28	10.25 ± 6.70	<0.001
CES-D (categories)				<0.001
None/Mild	10,848 (72.34)	10,481 (72.74)	367 (62.52)	
Moderate	3,340 (22.27)	3,176 (22.04)	164 (27.94)	
Severe	807 (5.38)	751 (5.21)	56 (9.54)	

Data are presented as mean ± standard deviations, number (proportion %). Missing data: Sex = 20, Education = 7, Marital status = 2, Residence = 892, BMI = 2,468, Smoking status = 3, Alcohol consumption = 3, Hypertension = 57, Diabetes = 95, Kidney disease = 50, Dyslipidemia = 224.

### Cross-sectional association between depressive symptoms and chronic liver disease

The multivariate logistic regression analysis showed that more depressive symptoms were associated with CLD (OR, 1.04; 95% CI,1.03, 1.06). Compared to participants with none/mild depressive symptoms, the ORs (95% CIs) of incident CLD were 1.46 (1.16, 1.83) and 1.78 (1.23, 2.56) for participants with moderate and severe depressive symptoms, respectively (Table 2).

**Table 2:** Odds ratio (OR) and 95% CIs (confidence intervals) for the cross-sectional association between depressive symptoms with chronic liver disease (CLD): results from logistic regression model.

Depressive symptoms	OR (95% CI)		
	Model 1^a^	Model 2^b^	Model 3^c^
Continuous	1.06 (1.04, 1.07)	1.06 (1.04, 1.07)	1.04 (1.03, 1.06)
Categorical			
None/Mild	Reference	Reference	Reference
Moderate	1.63 (1.34, 2.00)	1.65 (1.32, 2.05)	1.46 (1.16, 1.83)
Severe	2.53 (1.87, 3.44)	2.29 (1.62, 3.25)	1.78 (1.23, 2.56)

^a^Model 1 was adjusted for age, sex, education, marital status, residence. ^b^Model 2 was adjusted for BMI, smoking, alcohol consumption and variables in model 1. ^c^Model 3 was adjusted for hypertension, diabetes, stroke, heart disease, kidney disease, dyslipidemia, and variables in model 2.

### Characteristics of the longitudinal study population

A total of 13,405 participants (53.64% female; mean age: 58.58 ± 9.69 years) were included in the longitudinal analysis. Compared to participants with none/mild depressive symptoms, those with depressive symptoms were more likely to be older, female, non-smokers, non-drinkers and living in rural, and have lower education and BMI, other marital status, hypertension, diabetes, kidney disease, and higher CES-D (Table 3).

**Table 3:** Baseline characteristics of the longitudinal study population by depressive symptoms’ categories (*n* = 13,405).

Characteristics	Depressive symptoms			
	None/Mild	Moderate	Severe	*P*
	N = 9,783	N = 2,933	N = 689	
Age (years)	58.08 ± 9.57	59.75 ± 9.92	60.72 ± 9.72	<0.001
Female	4,860 (49.68)	1846 (62.94)	485 (70.39)	<0.001
Education level				<0.001
Illiterate	2,262 (23.12)	968 (33.00)	316 (45.86)	
Primary school and below	3,737 (38.20)	1,328 (45.28)	276 (40.06)	
Middle school and above	3,780 (38.64)	637 (21.72)	97 (14.08)	
Marital status				<0.001
Married	927 (9.48)	482 (16.43)	151 (21.92)	
Other	8,856 (90.52)	2,451 (83.57)	538 (78.08)	
Residence				<0.001
Rural	5,534 (56.57)	2006 (68.39)	519 (75.33)	
Urban	3,857 (39.43)	803 (27.38)	150 (21.77)	
BMI	23.72 ± 3.90	23.10 ± 3.85	23.07 ± 4.00	<0.001
Smoking status				<0.001
Never	5,804 (59.33)	1919 (65.43)	478 (69.38)	
Ever smoker	863 (8.82)	233 (7.94)	44 (6.39)	
Current smoker	3,113 (31.82)	781 (26.63)	167 (24.24)	
Alcohol consumption				<0.001
Never drinker	5,646 (57.71)	1894 (64.58)	450 (65.31)	
Former drinker	702 (7.18)	266 (9.07)	72 (10.45)	
Current drinker	3,434 (35.10)	772 (26.32)	167 (24.24)	
Hypertension	2,209 (22.58)	772 (26.32)	187 (27.14)	<0.001
Diabetes	485 (4.96)	186 (6.34)	55 (7.98)	0.001
Kidney disease	434 (4.44)	244 (8.32)	86 (12.48)	<0.001
Dyslipidemia	834 (8.52)	287 (9.79)	65 (9.43)	0.110
CES-D (continuous)	5.09 ± 3.31	15.10 ± 2.47	23.49 ± 2.35	<0.001

Data are presented as mean ± standard deviations, number (proportion %). Missing data: Residence = 536, BMI = 2042, Hypertension = 49, Diabetes = 88, Kidney disease = 37, Dyslipidemia = 200.

### Longitudinal association between depressive symptoms and chronic liver disease

During the follow-up (1,011 participants were followed up for 2 years, 1,467 participants were followed up for 4 years and 12,054 participants were followed up for 7 years, accounting for 84,379 person-years), 578 developed CLD, and the incident density of CLD was 6.85/1000 person-year. The multi-adjusted Cox regression model showed that more depressive symptoms were associated with CLD (HR, 1.04; 95% CI,1.02, 1.05), and each point increases of CES-D was related to a 4% higher risk of CLD. Compared to participants with none/mild depressive symptoms, the HRs (95% CIs) of incident CLD were 1.65 (1.33, 2.03) and 1.80 (1.24, 2.60) for those with moderate and severe depressive symptoms, respectively (Table 4).

**Table 4:** Harzads ratios (HRs) and 95% CIs (confidence intervals) and 50th percentile differences (PDs) in years of incident chronic liver disease (CLD) in the longitudinal relation to depressive symptoms: results from Cox regression model and Laplace regression model.

Depressive symptoms	HR (95% CI)			50th PDs (years) (95% CI)		
	Model 1^a^	Model 2^b^	Model 3^c^	Model 1^a^	Model 2^b^	Model 3^c^
Continuous	1.04 (1.02, 1.05)	1.04 (1.02, 1.05)	1.04 (1.02, 1.05)	−0.06 (−0.08, −0.04)	−0.06 (−0.09, −0.04)	−0.06 (−0.08, −0.03)
Categorical						
None/Mild	Reference	Reference	Reference	Reference	Reference	Reference
Moderate	1.62 (1.34, 1.96)	1.71 (1.39, 2.10)	1.65 (1.33, 2.03)	−0.83 (−1.15, −0.50)	−0.90 (−1.25, −0.56)	−0.83 (−1.18, −0.49)
Severe	1.98 (1.44, 2.73)	1.97 (1.38, 2.82)	1.80 (1.24, 2.60)	−1.16 (−1.70, −0.61)	−1.13 (−1.72, −0.53)	−0.96 (−1.56, −0.35)

^a^Model 1 was adjusted for age, sex, education, marital status, residence. ^b^Model 2 was adjusted for BMI, smoking, alcohol consumption and variables in model 1. ^c^Model 3 was adjusted for hypertension, diabetes, stroke, heart disease, kidney disease, dyslipidemia, and variables in model 2.

Laplace regression analysis showed that each score increases in depressive symptoms led to a 0.06-year earlier onset of CLD. The multi-adjusted 50th PDs (95% CI) of time (years) at incident CLD for the participants with moderate and severe depressive symptoms were, respectively, 0.83 (0.49, 1.18) and 0.96 (0.35, 1.56) years earlier by comparison to those with none/mild depressive symptoms (Table 4).

### Sensitivity analysis

In the cross-sectional analysis (Supplementary Table 1), participants with the data of covariates were more likely to be female, married, smokers, drinkers, living in rural, and have significantly lower education, hypertension, diabetes, kidney disease and dyslipidemia than those lacking the data. The difference between the two groups in age, BMI, CES-D, and CLD was not significant. In the longitudinal analysis (Supplementary Table 2), participants with the data of covariates were more likely to be married, smokers, drinkers, living in rural, and have significantly lower education, hypertension, diabetes, kidney disease and dyslipidemia than those lacking the data. The difference between the two groups in age, gender, BMI, CES-D, and CLD was not significant. The results were not altered much compared with those from the initial analyses when missing data were imputed by multiple imputation (Supplementary Tables 3, 4). In the stratified analyses, the associations of moderate depressive symptoms with CLD were similar between groups in males and females, smokers and non-smokers, and BMI < 24 kg/m^2^ and BMI ≥ 24 kg/m^2^. While the associations of severe depressive symptoms with CLD were not significant between these groups, which might be explained by the fewer cases in these groups (Supplementary Tables 5–7). The association between depressive symptoms and incident CLD was more pronounced among non-drinkers (HR, 2.10; 95% CI, 1.39–3.19), compared to that among drinkers (HR, 1.16; 95% CI, 0.51–2.68; Supplementary Table 8). The association between depressive symptoms and incident CLD was significant among the married (HR, 2.04; 95% CI, 1.37–3.03), but not in people with other marital status (HR, 1.00; 95% CI, 0.35–2.89; Supplementary Table 9). While the multiplicative interaction of depressive symptoms with alcohol consumption (*P* for interaction = 0.195) and marital status (*P* for interaction = 0.087) was not significant.

## Discussion

This study firstly evaluated the cross-sectional and longitudinal association of depressive symptoms with CLD based on a nationally representative cohort study in China. We found that more depressive symptoms were associated with a higher prevalence of CLD in cross-sectional analysis. In the longitudinal analysis, higher depressive symptoms were related to an increased risk of CLD. In addition, patients with moderate or severe depressive symptoms developed CLD 0.83 years and 0.96 years earlier than those with none/mild depressive symptoms.

Some cross-sectional studies have investigated the association between depressive symptoms and CLD in general or special populations. Among 766,427 adults in the general population in Taiwan (10, 19), Hsu Jer-Hwa et al. found significant differences in the degree of association between depressive symptoms and CLD in different states. The incidence of CLD in the population with bipolar disorder was 1.71 times that in people without bipolar disorder (19). In another study, the prevalence of CLD in patients with major depression was 2.27 times that in the general population without major depression (10). In addition, a study conducted in Beijing, China (20), found among the hospitalized patients with mental disorders (30.51% of them had depressive symptoms) that the prevalence of CLD was very common. However, few studies have focused on the longitudinal relationship in middle-aged and older Chinese. Our study found that higher depressive symptoms were not only associated with a higher prevalence of CLD at baseline but also associated with a higher risk of CLD during the follow-up and could further shorten the onset time of CLD, using a nationally representative sample in mainland China.

The mechanism by which depressive symptoms increase the risk of CLD morbidity is unclear. Several factors could be considered when interpreting the results. First, some risk behaviors tend to be more common in patients with depressive symptoms (21), such as less exercise, higher calorie diets, and smoking; each of these may increase the incidence likelihood of CLD. Second, mental stress and psychological stress also contribute to a greater likelihood of CLD. Severe depressive symptoms will increase mental pressure and psychological stress, making the nervous, endocrine and other systems in a state of high tension (22–24). Then these physiological reactions may affect the physiological function of the liver, promoting the occurrence and development of liver disease (14, 25). Finally, depressive symptoms and anxiety could increase the risk of infection and reactivation of latent infection for chronic hepatitis B virus. According to the report (26, 27), depressive symptoms have been shown to increase IL-10, which is thought to trigger a feedback mechanism that dampens liver inflammation. And IL-10 also leads to the inhibition of HBV-specific CD8 T cell responses. Therefore, patients with depressive symptoms may have a reduced ability to clear pathogens, increasing the risk of HBV flares.

Our study used a national cohort with relatively long follow-up periods to investigate the cross-sectional and longitudinal association of depressive symptoms with CLD. However, several limitations in this study include: (1) the associations between depressive symptoms and some specific CLDs were not reported as the data about CLDs was not subdivided. (2) The detailed depressive symptoms with respect to CLD were not discussed in this study. (3) CLD was assessed by retrospective self-report, which may result in recall bias. (4) Previous studies have suggested that experiencing liver disease were more likely to have impaired psychopathological profile including depressive symptoms (28, 29). Thus, it may be a limitation that the bidirectionality of relationship between CLD and depression symptoms did not considered. (5) Lacking the data on the gravity of CLD resulted in our inability to analyze the relationship between depressive symptoms and gravity of CLD, which maybe a limitation.

In conclusion, we found that more depressive symptoms could be associated with a higher prevalence of CLD and an increased risk of CLD and shorten the onset time of CLD in middle-aged and older Chinese adults. Our findings underscore the importance of maintaining good mental health for liver health. To reduce the risk of CLD, effective treatment and psychosocial interventions could be implemented for patients with depressive symptoms.

## Data availability statement

The original contributions presented in the study are included in the article/Supplementary materials, further inquiries can be directed to the corresponding authors.

## Ethics statement

The studies involving humans were approved by the Institutional Review Board of Peking University (IRB00001052-11015). The studies were conducted in accordance with the local legislation and institutional requirements. The participants provided their written informed consent to participate in this study.

## Author contributions

HX: Conceptualization, Project administration, Resources, Supervision, Writing – review & editing. JZ: Data curation, Formal analysis, Methodology, Writing – original draft, Writing – review & editing. XL: Data curation, Formal analysis, Methodology, Writing – original draft, Writing – review & editing. SW: Data curation, Methodology, Writing – original draft, Writing – review & editing. DZ: Data curation, Methodology, Writing – original draft, Writing – review & editing. JY: Data curation, Methodology, Writing – review & editing. CH: Data curation, Methodology, Writing – review & editing. ML: Data curation, Methodology, Writing – review & editing. WZ: Conceptualization, Project administration, Resources, Supervision, Writing – review & editing.
