# Supplementary material for: Association of depressive symptoms with chronic liver disease among middle-aged and older adults in China
Source: Front Psychiatry. 2023 Oct 24;14:1273754. doi: 10.3389/fpsyt.2023.1273754 (PMC10628464; doi:10.3389/fpsyt.2023.1273754)
Supplement: Supplementary file 1 [file Table_1.DOCX]

**Supplementary Table 1.** Characteristics between individuals having the data of covariates and those lacking the data in the cross-sectional study.

| **Characteristics** | Total | Without data | With data | *P* |
| --- | --- | --- | --- | --- |
|  | N=14,995 | N=3,374 | N=11,621 |  |
| Age (years) | 58.85±9.87 | 59.00±10.68 | 58.81±9.62 | 0.31 |
| Female | 7,962 (53.10) | 1,755 (52.02) | 6,207 (53.41) | <0.001 |
| Education level |  |  |  | <0.001 |
| Illiterate | 3,944 (26.30) | 846 (25.07) | 3,098 (26.66) |  |
| Primary school and below | 5,947 (39.66) | 1,149 (34.05) | 4,798 (41.29) |  |
| Middle school and above | 5,097 (33.99) | 1,372 (40.66) | 3,725 (32.05) |  |
| Marital status |  |  |  | <0.001 |
| Other | 1,818 (12.12) | 332 ( 9.84) | 1,486 (12.79) |  |
| Married | 13,175 (87.86) | 3,040 (90.10) | 10,135 (87.21) |  |
| Residence |  |  |  | <0.001 |
| Rural | 8,751 (58.36) | 1,229 (36.43) | 7,522 (64.73) |  |
| Urban | 5,352 (35.69) | 1,253 (37.14) | 4,099 (35.27) |  |
| BMI | 23.53±3.91 | 23.57±4.15 | 23.52±3.89 | 0.76 |
| Smoking status |  |  |  | <0.001 |
| Never | 9,120 (60.82) | 2,124 (62.95) | 6,996 (60.20) |  |
| Ever smoker | 1,342 ( 8.95) | 315 ( 9.34) | 1,027 ( 8.84) |  |
| Current smoker | 4,530 (30.21) | 932 (27.62) | 3,598 (30.96) |  |
| Alcohol consumption |  |  |  | 0.002 |
| Never drinker | 8,912 (59.43) | 2,034 (60.28) | 6,878 (59.19) |  |
| Former drinker | 1,234 ( 8.23) | 294 ( 8.71) | 940 ( 8.09) |  |
| Current drinker | 4,846 (32.32) | 1,043 (30.91) | 3,803 (32.73) |  |
| Hypertension | 3,651 (24.35) | 921 (27.30) | 2,730 (23.49) | <0.001 |
| Diabetes | 856 ( 5.71) | 198 ( 5.87) | 658 ( 5.66) | <0.001 |
| Kidney disease | 947 ( 6.32) | 226 ( 6.70) | 721 ( 6.20) | <0.001 |
| Dyslipidemia | 1,386 ( 9.24) | 353 (10.46) | 1,033 ( 8.89) | <0.001 |
| CES-D (continuous) | 8.33±6.31 | 8.24±6.39 | 8.36±6.28 | 0.33 |
| CES-D (tertiles) |  |  |  | 0.62 |
| None/Mild | 10,848 (72.34) | 2,441 (72.35) | 8,407 (72.34) |  |
| Moderate | 3,340 (22.27) | 741 (21.96) | 2,599 (22.36) |  |
| Severe | 807 ( 5.38) | 192 ( 5.69) | 615 ( 5.29) |  |
| Chronic liver disease | 587 (3.91) | 145 (4.30) | 442 (3.80) | 0.190 |

Data are presented as mean ± standard deviations, number (proportion %).

Missing data: Education=7, Marital status=2, Residence=892, BMI=2468, Smoking status=3, Alcohol consumption=3, Hypertension=57, Diabetes=95, Kidney disease=50, Dyslipidemia=224.

**SupplementaryTable 2.** Characteristics between individuals having the data of covariates and those lacking the data in the longitudinal study..

| **Characteristics** | Total | Without data | With data | *P* |
| --- | --- | --- | --- | --- |
|  | N=13,405 | N=2,665 | N=10,740 |  |
| Age (years) | 58.58±9.69 | 58.52±10.46 | 58.60±9.49 | 0.68 |
| Female | 7,191 (53.64) | 1,407 (52.80) | 5,784 (53.85) | 0.33 |
| Education level |  |  |  | <0.001 |
| Illiterate | 3,546 (26.45) | 661 (24.80) | 2,885 (26.86) |  |
| Primary school and below | 5,341 (39.84) | 903 (33.88) | 4,438 (41.32) |  |
| Middle school and above | 4,514 (33.67) | 1,097 (41.16) | 3,417 (31.82) |  |
| Marital status |  |  |  | <0.001 |
| Other | 1,560 (11.64) | 258 ( 9.68) | 1,302 (12.12) |  |
| Married | 11,845 (88.36) | 2,407 (90.32) | 9,438 (87.88) |  |
| Residence |  |  |  | <0.001 |
| Rural | 8,059 (60.12) | 1,087 (40.79) | 6,972 (64.92) |  |
| Urban | 4,810 (35.88) | 1,042 (39.10) | 3,768 (35.08) |  |
| BMI | 23.55±3.90 | 23.67±4.02 | 23.54±3.90 | 0.42 |
| Smoking status |  |  |  | <0.001 |
| Never | 8,201 (61.18) | 1,695 (63.60) | 6,506 (60.58) |  |
| Ever smoker | 1,140 ( 8.50) | 233 ( 8.74) | 907 ( 8.45) |  |
| Current smoker | 4,061 (30.29) | 734 (27.54) | 3,327 (30.98) |  |
| Alcohol consumption |  |  |  | 0.015 |
| Never drinker | 7,990 (59.60) | 1,610 (60.41) | 6,380 (59.40) |  |
| Former drinker | 1,040 ( 7.76) | 216 ( 8.11) | 824 ( 7.67) |  |
| Current drinker | 4,373 (32.62) | 837 (31.41) | 3,536 (32.92) |  |
| Hypertension | 3,168 (23.63) | 718 (26.94) | 2,450 (22.81) | <0.001 |
| Diabetes | 726 ( 5.42) | 145 ( 5.44) | 581 ( 5.41) | <0.001 |
| Kidney disease | 764 ( 5.70) | 163 ( 6.12) | 601 ( 5.60) | <0.001 |
| Dyslipidemia | 1,186 ( 8.85) | 276 (10.36) | 910 ( 8.47) | <0.001 |
| CES-D (continuous) | 8.23±6.25 | 8.09±6.29 | 8.26±6.24 | 0.22 |
| CES-D (tertiles) |  |  |  | 0.81 |
| None/Mild | 9,783 (72.98) | 1,949 (73.13) | 7,834 (72.94) |  |
| Moderate | 2,933 (21.88) | 574 (21.54) | 2,359 (21.96) |  |
| Severe | 689 ( 5.14) | 142 ( 5.33) | 547 ( 5.09) |  |
| Chronic liver disease | 578 ( 4.31) | 120 ( 4.50) | 458 ( 4.26) | 0.59 |

Data are presented as mean ± standard deviations, number (proportion %).

Missing data: Education=4, Marital status=35, Residence=536, BMI=2042, Smoking status=3, Alcohol consumption=2, Hypertension=49, Diabetes=88, Kidney disease=37, Dyslipidemia=200.

**Supplementary Table 3.** Odds ratio (OR) and 95% CIs (confidence intervals) for the cross-sectional association between depressive symptoms with chronic liver disease (CLD) with multiple imputation for missing data: results from Logistic regression model.

| **Depressive symptoms** | **OR (95% CI)** | | |
| --- | --- | --- | --- |
|  | Model 1^a^ | Model 2^b^ | Model 3^c^ |
| Continuous | 1.06 (1.04, 1.07) | 1.06 (1.04, 1.07) | 1.04 (1.03, 1.06) |
| Categorical |  |  |  |
| None/Mild | Reference | Reference | Reference |
| Moderate | 1.59 (1.31,1.94) | 1.61 (1.33,1.96) | 1.41 (1.15,1.71) |
| Severe | 2.43 (1.81,3.28) | 1.78 (1.23, 2.56) | 1.89 (1.39,2.57) |

^a^ Model 1 was adjusted for age, sex, education, marital status, residence.

^b^ Model 2 was adjusted for BMI, smoking, alcohol consumption and variables in model 1.

^c^ Model 3 was adjusted for hypertension, diabetes, stroke, heart disease, kidney disease, dyslipidemia, and variables in model 2.

**Supplementary Table 4.** Harzads ratios (HRs) and 95% CIs (confidence intervals) and 50th percentile differences (PDs) in years of incident chronic liver disease (CLD) in the longitudinal relation to depressive symptoms with multiple imputation for missing data : results from Cox regression model and Laplace regression model.

| **Depressive Symptoms** | **HR (95% CI)** | | |  | **50th PDs (years) (95% CI)** | | |
| --- | --- | --- | --- | --- | --- | --- | --- |
|  | Model 1^a^ | Model 2^b^ | Model 3^c^ |  | Model 1^a^ | Model 2^b^ | Model 3^c^ |
| Continuous | 1.04 (1.02, 1.05) | 1.04 (1.03, 1.05) | 1.04 (1.02, 1.05) |  | -0.06 (-0.08, -0.04) | -0.06 (-0.09, -0.04) | -0.06 (-0.08, -0.04) |
| Categorical |  |  |  |  |  |  |  |
| None/Mild | Reference | Reference | Reference |  | Reference | Reference | Reference |
| Moderate | 1.62 (1.34, 1.96) | 1.65 (1.37, 1.99) | 1.57(1.30,1.90) |  | -0.80(-1.12,-0.49) | -0.84(-1.15,-0.52) | -0.76(-1.07,-0.44) |
| Severe | 1.95 (1.42, 2.69) | 1.99(1.44,2.74) | 1.85(1.34,2.55) |  | -1.10(-1.63,-0.58) | -1.14(-1.67,-0.61) | -1.01(-1.55,-0.48) |

^a^ Model 1 was adjusted for age, sex, education, marital status, residence.

^b^ Model 2 was adjusted for BMI, smoking, alcohol consumption and variables in model 1.

^c^ Model 3 was adjusted for hypertension, diabetes, stroke, heart disease, kidney disease, dyslipidemia, and variables in model 2.

**Supplementary Table 5.** Harzads ratios (HRs) and 95% CIs (confidence intervals) and 50th percentile differences (PDs) in years of incident chronic liver disease (CLD) in the longitudinal relation to depressive symptoms by sex: results from Cox regression model and Laplace regression model.

| **Depressive Symptoms** | **HR (95% CI)** ^a^ | | | |  | **50th PDs (years) (95% CI)** ^a^ | |
| --- | --- | --- | --- | --- | --- | --- | --- |
|  | n | Male | n | Female |  | Male | Female |
| Continuous | 4956 | 1.02(1.00, 1.04) | 5784 | 1.05(1.03, 1.07) |  | -0.03(-0.07, 0) | -0.07(-0.1, -0.04) |
| Categorical |  |  |  |  |  |  |  |
| None/Mild | 3920 | Reference | 3914 | Reference |  | Reference | Reference |
| Moderate | 871 | 1.60(1.17, 2.18) | 1488 | 1.71(1.28, 2.28) |  | -0.79(-1.31, -0.27) | -0.84(-1.15, -0.52) |
| Severe | 165 | 1.52(0.79, 2.89) | 382 | 1.99(1.26, 3.15) |  | -0.72(-1.77, 0.34) | -1.14(-1.67, -0.61) |

^a^ Adjusted for age, sex, education, marital status, residence, BMI, smoking, alcohol consumption hypertension, diabetes, stroke, heart disease, kidney disease, and dyslipidemia.

**Supplementary Table 6.** Harzads ratios (HRs) and 95% CIs (confidence intervals) and 50th percentile differences (PDs) in years of incident chronic liver disease (CLD) in the longitudinal relation to depressive symptoms by smoking: results from Cox regression model and Laplace regression model.

| **Depressive Symptoms** | **HR (95% CI)** ^a^ | | | |  | **50th PDs (years) (95% CI)** ^a^ | |
| --- | --- | --- | --- | --- | --- | --- | --- |
|  | n | Non-smoker | n | Smoker |  | Non-drinker | Drinker |
| Continuous | 7413 | 1.04(1.02, 1.06) | 3327 | 1.03(1.00, 1.05) |  | -0.06(-0.09, -0.04) | -0.04(-0.08, 0) |
| Categorical |  |  |  |  |  |  |  |
| None/Mild | 5292 | Reference | 2542 | Reference |  | Reference | Reference |
| Moderate | 1708 | 1.64(1.27, 2.11) | 651 | 1.69(1.14, 2.49) |  | -0.84(-1.27, -0.42) | -0.81(-1.41, -0.22) |
| Severe | 413 | 1.98(1.31, 3.00) | 134 | 1.32(0.57, 3.04) |  | -1.14(-1.84, -0.43) | -0.46(-1.73, 0.8) |

^a^ Adjusted for age, sex, education, marital status, residence, BMI, smoking, alcohol consumption hypertension, diabetes, stroke, heart disease, kidney disease, and dyslipidemia.

**Supplementary Table 7.** Harzads ratios (HRs) and 95% CIs (confidence intervals) and 50th percentile differences (PDs) in years of incident chronic liver disease (CLD) in the longitudinal relation to depressive symptoms by BMI: results from Cox regression model and Laplace regression model.

| **Depressive Symptoms** | **HR (95% CI)** ^a^ | | | |  | **50th PDs (years) (95% CI)** ^a^ | |
| --- | --- | --- | --- | --- | --- | --- | --- |
|  | n | BMI<24 kg/m^2^ | n | BMI≥24 kg/m^2^ |  | BMI<24 kg/m^2^ | BMI≥24 kg/m^2^ |
| Continuous | 4351 | 1.03(1.01,1.05) | 6389 | 1.04(1.02,1.06) |  | -0.05(-0.08,-0.01) | -0.06(-0.1,-0.03) |
| Categorical |  |  |  |  |  |  |  |
| None/Mild | 3300 | Reference | 4534 | Reference |  | Reference | Reference |
| Moderate | 862 | 1.59(1.16,2.19) | 1497 | 1.67(1.26,2.22) |  | -0.79(-1.33, -0.25) | -0.86(-1.31,-0.4) |
| Severe | 189 | 1.35(0.72,2.51) | 358 | 2.19(1.38,3.47) |  | -0.46(-1.54, 0.61) | -1.27(-2,-0.53) |

^a^ Adjusted for age, sex, education, marital status, residence, BMI, smoking, alcohol consumption hypertension, diabetes, stroke, heart disease, kidney disease, and dyslipidemia.

**Supplementary Table 8.** Harzads ratios (HRs) and 95% CIs (confidence intervals) and 50th percentile differences (PDs) in years of incident chronic liver disease (CLD) in the longitudinal relation to depressive symptoms by alcohol consumption: results from Cox regression model and Laplace regression model.

| **Depressive Symptoms** | **HR (95% CI)** ^a^ | | | |  | **50th PDs (years) (95% CI)** ^a^ | |
| --- | --- | --- | --- | --- | --- | --- | --- |
|  | n | Non-drinker | n | Drinker |  | Non-drinker | Drinker |
| Continuous | 7204 | 1.04(1.02, 1.06) | 3536 | 1.02(0.99, 1.05) |  | -0.07(-0.10, -0.04) | -0.03(-0.07, 0.01) |
| Categorical |  |  |  |  |  |  |  |
| None/Mild | 5058 | Reference | 2776 | Reference |  | Reference | Reference |
| Moderate | 1733 | 1.79(1.39, 2.30) | 626 | 1.37(0.92, 2.04) |  | -1.00(-1.43, -0.57) | -0.49(-1.10, 0.13) |
| Severe | 413 | 2.10(1.39, 3.19) | 134 | 1.16(0.51, 2.68) |  | -1.24(-1.95, -0.52) | -0.26(-1.55, 1.04) |

^a^ Adjusted for age, sex, education, marital status, residence, BMI, smoking, alcohol consumption hypertension, diabetes, stroke, heart disease, kidney disease, and dyslipidemia.

**Supplementary Table 9.** Harzads ratios (HRs) and 95% CIs (confidence intervals) and 50th percentile differences (PDs) in years of incident chronic liver disease (CLD) in the longitudinal relation to depressive symptoms by married status: results from Cox regression model and Laplace regression model.

| **Depressive Symptoms** | **HR (95% CI)** ^a^ | | | |  | **50th PDs (years) (95% CI)** ^a^ | |
| --- | --- | --- | --- | --- | --- | --- | --- |
|  | n | Married | n | Others |  | Married | Others |
| Continuous | 11845 | 1.04(1.02, 1.05) | 1506 | 1.02(0.98,1.07) |  | -0.06(-0.09,-0.04) | -0.04(-0.11,0.02) |
| Categorical |  |  |  |  |  |  |  |
| None/Mild | 8856 | Reference | 927 | Reference |  | Reference | Reference |
| Moderate | 2451 | 1.74(1.39, 2.18) | 482 | 1.21 (0.66,2.23) |  | -0.92(-1.29, -0.55) | -0.35(-1.28,0.58) |
| Severe | 538 | 2.04(1.37, 3.03) | 151 | 1.00 (0.35, 2.89) |  | -1.16(-1.80, -0.53) | -0.52(-1.82,1.72) |

^a^ Adjusted for age, sex, education, marital status, residence, BMI, smoking, alcohol consumption hypertension, diabetes, stroke, heart disease, kidney disease, and dyslipidemia.
